# Antimicrobial Resistance Profile, Whole-Genome Sequencing and Core Genome Multilocus Sequence Typing of *B. anthracis* Isolates in Croatia from 2001 to 2022

**DOI:** 10.3390/antibiotics13070639

**Published:** 2024-07-11

**Authors:** Gordan Kompes, Sanja Duvnjak, Irena Reil, Željko Mihaljević, Boris Habrun, Miroslav Benić, Luka Cvetnić, Silvio Špičić, Antonela Bagarić

**Affiliations:** 1 Laboratory for General Bacteriology and Mycology, Department for Bacteriology and Parasitology, Croatian Veterinary Institute, 10000 Zagreb, Croatia; kompes@veinst.hr (G.K.); habrun@veinst.hr (B.H.); nela.marijan@gmail.com (A.B.); 2 Laboratory for Bacterial Zoonoses and Molecular Diagnostics of Bacterial Diseases, Department for Bacteriology and Parasitology, Croatian Veterinary Institute, 10000 Zagreb, Croatia; spicic@veinst.hr; 3 Laboratory for Pathology, Department for Pathology, Croatian Veterinary Institute, 10000 Zagreb, Croatia; miha@veinst.hr; 4 Laboratory for Mastitis and Raw Milk Quality, Department for Bacteriology and Parasitology, Croatian Veterinary Institute, 10000 Zagreb, Croatia; benic@veinst.hr (M.B.); lcvetnic@veinst.hr (L.C.)

**Keywords:** *B. anthracis*, antimicrobial resistance, WGS, cgMLST

## Abstract

*Bacillus anthracis*, the causative agent of anthrax disease, is a worldwide threat to livestock, wildlife and public health. It is also considered one of the most important pathogens of bioterrorism. Rapid and reliable diagnosis and administration of antimicrobials are essential for effective anthrax treatment. In this study, we determined the in vitro susceptibilities of 40 isolates of *B. anthracis* isolated in Croatia over the recent two decades to 18 antimicrobials. Whole-genome sequencing was performed, and bioinformatics tools were used to determine virulence factors and antimicrobial resistance genes. Core genome-based multilocus sequence typing was used for isolate comparison and phylogenetic analysis. All isolates were susceptible to all antimicrobials recommended for post-exposure prophylaxis or anthrax therapy. Susceptibility was found to all other tested antimicrobials that are an alternative for primary therapy. We found two beta-lactamase genes, but their expression is not sufficient to confer resistance. In all isolates used in this study, we found 21 virulence genes, 8 of which are responsible for toxin and capsule production. As far as phylogenetic analysis is concerned, the *B. anthracis* isolates from Croatia are categorised into two clades. The first is clade A, subclade Trans Eurasia, and the other is clade B, subclade B2.

## 1. Introduction

*Bacillus* (*B.*) *anthracis*, the causative agent of anthrax, belongs to the Bacillus cereus group together with at least six other species (*B. cereus*, *B. thuringiensis*, *B. mycoides*, *B. pseudomycoides*, *B. weihenstephanensis* and *B. cytotoxicus*). They are Gram-positive, spore-forming, aerobic, facultatively anaerobic, rod-shaped bacteria [1]. In contrast to other Bacillus species, it is non-motile, non-haemolytic on sheep blood agar, grows at a temperature of 37 °C, and forms large colonies with irregularly tapered outgrowths. In culture, it tends to form long chains of bacteria. Within a host, however, it appears either as single organisms or as chains consisting of two or three bacilli [2]. Anthrax is primarily a disease of herbivores, although it has also been reported to occur in omnivores, carnivores, and other vertebrates. Herbivorous animals, such as cattle, sheep, and goats, ingest spores in the soil and become infected. Humans can become infected through direct contact with infected animals or tissue from infected animals or through direct exposure to *B. anthracis* [3]. Based on the initial route of infection, three primary forms of clinical infection in humans are described: inhalational anthrax, gastrointestinal anthrax, and cutaneous anthrax [4] A new form of soft tissue infection associated with injection drug use, termed injectional anthrax, has also been described [5]. 

*B. anthracis* is also considered one of the most important pathogens of bioterrorism. Due to the often-fatal outcome of human cases, the rapid administration of clearly effective antimicrobials is critical, whether for prophylaxis, following suspected exposure, or for the treatment of clinical cases. *B. anthracis* is a bacterium that is sensitive to most antibiotics. However, early treatment is critical to eliminate the bacterium before it releases toxins into the bloodstream [6,7]. 

When conditions are not favourable for the growth and multiplication of vegetative forms of *B. anthracis*, they begin to form spores. Spores are highly resistant to biological extremes such as heat, cold, pH, desiccation, chemicals, irradiation, and other unfavourable conditions. The organism can remain in the spore stage for long periods of time, waiting for the moment when conditions are favourable for germination and multiplication [8]. Jacotot and Virat [9] and Wilson and Russell [10] succeeded in reviving spores after more than 60 years, while de Vos [11] recovered anthrax spores from bones after 200 ± 50 years. From the earliest historical records until the development of an effective veterinary vaccine in the middle of the 20th century and the subsequent advent of antibiotics, the disease was a major cause of uncontrolled mortality in cattle, sheep, goats, horses, and pigs worldwide [8]. Anthrax is found all over the world, on every continent except Antarctica. There are endemic areas with more frequent outbreaks, while sporadic outbreaks occur in other areas [8,12,13]. *B. anthracis* harbours two plasmids, pXO1 (182 kb) and pXO2 (95 kb). The most important virulence factors, the tripartite anthrax toxin genes *cya*, *lef*, and *pag*, are located on the plasmid pXO1 [14,15,16], while the poly-γ-D-glutamic acid capsule genes *capABCDE* are localised on the plasmid pXO2 [17,18]. Previously, the presence of these two virulence plasmids was considered the main distinguishing feature between *B. anthracis* and closely related species, particularly *Bacillus cereus* and *Bacillus mycoides* [19]. In the meantime, the virulence plasmids pXO1 and pXO2 have also been found in other isolates of the B. cereus group [20,21,22]. *B. anthracis* is an evolutionarily young pathogen characterised by a lack of molecular variation. Isolates from all over the world show an extremely high genetic homology [23,24]. It is assumed that the dormant state of its spores probably greatly reduces the rate of evolutionary change of *B. anthracis* and contributes to its extreme homogeneity [25]. Due to this lack of diversity, only modern molecular characterisation techniques have proven effective in distinguishing strains within this highly clonal species. Standard genotyping methods to determine the phylogenetic relationships between *B. anthracis* isolates include canonical single-nucleotide polymorphisms (canSNPs) and multilocus variable-number tandem repeat analysis (MLVA) [26,27,28]. Recently, core-genome-based multilocus sequence typing (cgMLST) has been increasingly used as a comparative tool using WGS data [29]. Phylogenetic analysis has defined three major clades, with C being basal to clades A and B. Members of clade A are most commonly observed worldwide (~90%), while members of clades B (~10%) and C (<1%) are much less frequent [27]. Clade A is divided into seven subclades: Ancient A, Vollum, V770, Sterne, Ames, Australia 94, and Trans Eurasia [30].

Historical data on anthrax in humans and animals in Croatia can be found in the Supplementary Materials.

Anthrax has occurred only sporadically in Croatia, as immunoprophylaxis was systematically carried out in enzootic areas where anthrax outbreaks had occurred [31]. Since 1981, a total of 59 cases of anthrax in animals have been confirmed at the Croatian Veterinary Institute in Croatia. The course of the disease is characterised by the fact that in some periods the disease does not occur. Within the period of more than four decades, no disease was detected in 20 years (1981–1983, 1992–1996, 1998–2000, 2008–2010, 2013, 2015–2019 and 2021) [31,32]. Only a few major outbreaks have been described in recent decades: 2001/2002 (11 animals–5 isolates) [33], 2006/2007 (11 animals–6 isolates) [34], and the largest outbreak in 2022 (323 animals–25 isolates) [35]. Sporadic cases occur mainly in the southern part of Croatia, where sporadic cases have been recorded over several years (4 isolates tested in this study) [31].

The aim of this study was to describe the reliable and rapid diagnosis of epizootic 2022 and to determine the in vitro antimicrobial resistance (AMR) of 40 isolates of *B. anthracis* isolated in Croatia in the recent two decades to 18 different antibiotics. In addition, we performed whole-genome sequencing (WGS) on all isolates to determine AMR and virulence genes and used cgMLST as a tool for isolate comparison and phylogenetic analysis.

## 2. Results

### 2.1. Isolates

A total of 40 isolates were included in this study. In total, 25 isolates were from the 2022 outbreak, while 15 isolates were obtained from laboratory strain collections from outbreaks in the previous years (2001/2002-5 isolates, 2006/2007-6 isolates) and from areas with sporadic anthrax occurrence (ST) (4 isolates). As shown in Table 1, the presence of the virulent plasmids pXO1 and pXO2 was confirmed in all 40 isolates. Strain Sterne 34F2 lacked the pXO2 plasmid.

### 2.2. Antimicrobial Susceptibility

The results—expressed as MIC at which 50% and 90% of the tested isolates were inhibited (MIC_50_, MIC_90_)—the range (mg/L), the breakpoint, and the percentage of susceptible isolates are shown in Table 2.

All isolates showed susceptibility to quinupristin/dalfopristin, vancomycin, ampicillin, rifampin, levofloxacin, penicillin, oxacillin + 2%NaCl, clindamycin, linezolid, tetracycline, gentamicin, gatifloxacin, and ciprofloxacin. However, 40% of the isolates were intermediate susceptible to ceftriaxone, while 5% were intermediate susceptible to erythromycin. All isolates tested were resistant to daptomycin and trimethoprim/sulfamethoxazole.

### 2.3. WGS and Bioinformatic Analysis

The genes for antimicrobial resistance and virulence factors, protein function, percentage of overlap, and identities found in 40 *B. anthracis* strains isolated in Croatia are listed in Table 3. 

We identified a total of four AMR genes in all *B. anthracis* isolates from the 2001/2002 outbreak and isolates from the southern part of Croatia (*bla*, *blaII*, *fosB*, *satA*), while isolates from the 2006/2007 and 2022 outbreaks lacked the *fosB* gene.

As far as the virulence genes are concerned, all genes listed in Table 3 were found in all tested isolates without exception.

### 2.4. Core-Genome-Based Multilocus Sequence Typing (cgMLST)

cgMLST analysis was performed for 3803 core loci and 1263 accessory genomic loci. Forty-seven and forty-five loci were missing in the 2001/2002 outbreak samples and in the samples from southern Croatia, respectively. Complex type 219 was defined for the samples from the 2001/2002 outbreak and 222 for the samples from the southern part of the country.

In total, 145 and 148 loci were missing in the samples from the 2006/2007 and 2022 outbreaks, respectively. Complex type 220 was defined for samples from 2006/2007 and 221 for samples from the 2022 outbreak.

**Figure 1:**
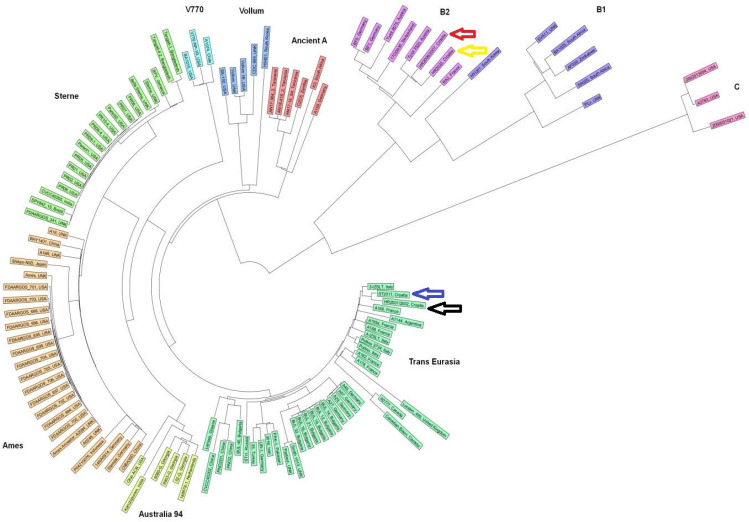
Phylogenetic analysis of the strains using a neighbour-joining tree based on the cgMLST data. The colours represent the clade and subclade, which are also indicated by the name, while the node labels correspond to the names of the strains and the country of origin. The isolates used in this study are indicated by arrows (red—outbreak 2006/2007; yellow—outbreak 2022; blue—isolates from southern part of Croatia; black—outbreak 2001/2002).

The phylogenetic analysis of the strains using a neighbour-joining tree cluster analysis based on the cgMLST data (Figure 1) shows that the *B. anthracis* isolates from the outbreaks in Croatia were divided into two clades. Isolates from the 2001/2002 outbreak and isolates from southern part of country were categorised in clade A, subclade Trans Eurasia, while isolates from the 2006/2007 and 2022 outbreaks were categorised in clade B, subclade B2.

## 3. Discussion

In Europe, anthrax is considered a sporadically occurring disease that almost exclusively affects animals at pastures [36]. In Croatia, anthrax is also rare in animals and humans, but there are sporadic outbreaks, especially in the anthrax districts, which are mainly located in the Lonjsko Polje region and in the southern parts of Croatia [31]. The situation is similar in the neighbouring countries where the occurrence of anthrax has been described: Italy [37], Slovenia [38], Hungary [39], and Bosnia and Herzegovina [40]. 

Antimicrobial susceptibility testing plays a key role in the treatment of anthrax infections. Rapid diagnosis is also crucial for post-exposure prophylaxis (PEP) or the early treatment of anthrax to eliminate the bacterium before it releases toxins into the bloodstream [6]. Beta-lactam antibiotics such as penicillin are recommended by the WHO and CDC as prophylactic therapy for anthrax, while tetracyclines and fluoroquinolones are recommended by the CDC for PEP and for the treatment of anthrax [8,41]. 

As shown in this study, a reliable diagnosis of anthrax can be made within 24 h based on colony morphology, haemolysis on blood agar, and the detection of the plasmids pXO1 and pXO2. In this study, a total of 40 *B. anthracis* strains isolated in Croatia were analysed for their susceptibility to antimicrobial agents. All tested isolates were susceptible to penicillin, tetracycline, and ciprofloxacin, with MIC_90_ of ≤0.06, ≤2, and ≤0.5, respectively. The first penicillin resistance in a clinical *B. anthracis* isolate was described in 1976 in a fatal case of anthrax in Northampton, England [42]. Although penicillin resistance of *B. anthracis* is thought to be at least 10% [43,44], the largest study which included 110 strains from outbreaks in Italy reported a 100% susceptibility to penicillin [45]. In this study, we found two beta-lactamase genes, *bla* and *blaII*. It has already been described that *B. anthracis* possesses these two beta-lactamase genes, *bla1* (penicillinase) and *bla2* (cephalosporinase), but that their transcription is very low and gene expression is not sufficient to confer resistance to beta-lactam antibiotics [46,47]. Although these genes are present, it has been hypothesised that they are not expressed due to a truncation of the positive regulatory gene, *plcR* [48]. The transcriptional activator PlcR is responsible for the expression of extracellular proteins such as haemolysins, enterotoxins, and proteases in *B. cereus* and *B. thuringiensis* [49,50]. The sequence of the *plcR* gene of *B. anthracis* strains indicates that the expression of the gene results in the synthesis of a truncated PlcR protein, in which codon 214 for glutamic acid (GAA or GAG) in *B. cereus* and *B. thuringiensis* is converted to a termination codon (TAA) in *B. anthracis* by a single nucleotide substitution (G→T) [51]. Although the *plcR* homologue of *B. anthracis* is truncated, there are 56 putative *plcR* binding motifs on the chromosome and 2 on pXO2. Extracellular protein genes include phospholipases, enterotoxins, and haemolysins, and the *plcR* mutation has been shown to be responsible for a drastic reduction in lecithinase, protease, and haemolysin production, giving *B. anthracis* a different secretome to *B. cereus* [48,52]. It has been shown that the transcription of beta-lactamases is controlled by an extracytoplasmic sigma factor (ECF) SigP and its anti-sigma factor RsiP [53]. Beta-lactamase expression in *B. cereus* and *B. thuringiensis* is inducible by beta-lactam antibiotics, leading to the proteolysis of RsiP, release of SigP, and transcription of the corresponding genes [54]. This beta-lactam feedback system is not functional in prototypic *B. anthracis* isolates. However, in a penicillin-resistant *B. anthracis* strains, the *rsiP* gene was nonsense mutated and the strain constitutively produced beta-lactamases [53].

All *B. anthracis* isolates in our study were also found to be susceptible to ampicillin and oxacillin +2%NaCl, with MIC_90_ less or equal to the minimum concentration tested (≤0.12; ≤0.25). This is consistent with previous publications reporting MICs for the other penicillin that closely matched the penicillin MIC [44]. 

Regarding other fluoroquinolones used in this study, all isolates were found to be susceptible to levofloxacin and gatifloxacin, with MIC_90_ values ≤0.25 and ≤1, respectively. Ciprofloxacin and tetracycline resistance of *B. anthracis* strains is very rare and is only described in a few publications [44]. The susceptibility data presented in this study support the current recommendations for the use of penicillin, tetracycline, and ciprofloxacin in PEP and anthrax threats. 

Forty percent of the isolates tested in this study were intermediate susceptible to ceftriaxone, while sixty percent were sensitive. This is in partial agreement with Manzulli et al. [45] and Cavallo et al. [43], who categorised all isolates as intermediate susceptible to ceftriaxone. In contrast to these studies, Jones et al. [55] reported all isolates as sensitive to ceftriaxone. Due to this variable susceptibility, non-penicillin beta-lactams are not recommended for the treatment of anthrax.

All *B. anthracis* isolated in this study were also susceptible to linezolid, rifampin, gentamycin, vankomycin, quinupristin/dalfopristin, and clindamycin. This is consistent with previous published studies [45,56,57].

Linezolid is used for the treatment of a variety Gram-positive infections. It is a bacteriostatic oxazolidinone with a unique mode of action that inhibits bacterial protein synthesis by preventing the formation of the 70S ribosomal complex [58]. For this reason, cross-resistance is unlikely. It is therefore a good alternative for resistant *B. anthracis* strains. Although all isolates were susceptible to rifampin and clindamycin, they are not considered a good alternative for anthrax treatment as resistance can easily be generated in vitro [59,60]. Nevertheless, rifampin, clyndamicin, and vancomycin together with fluoroquinolones are considered good candidates for the treatment of inhalational anthrax and meningitis [61]. Athamna et al. [62] found that quinupristin/dalfopristin killed *B. anthracis* the fastest of all antimicrobial agents tested.

Resistance to trimethoprim/sulfamethoxazole was detected in all tested isolates. This result was to be expected as intrinsic resistance to this antimicrobial agent has already been described [63]. All isolates in this study were also resistant to daptomycin with MIC_50_ and MIC_90_ of 4 mg/L. This result is consistent with previous publications [56,64].

Another AMR gene found in all isolates was *satA*, while the *fosB* gene was only found in the *B. anthracis* isolates from the 2001/2002 outbreak and isolates from sporadic outbreaks isolated in southern Croatia. *SatA* belongs to the streptothricin acetyltransferase gene family and confers resistance to nucleoside antibiotics, which are produced by actinomycetes in soil. It has already been described in *B. anthracis* and *B. subtilis* [65]. The *fosB* gene is a chromosomally derived thiol transferase and confers resistance to fosfomycin [48]. In their study, Bruce et al. [66] assumed that the detection of the *fosB* gene can be a clear prediction for the affiliation of the isolates to clade C. Clade C is largely defined by the absence of the *fosB* gene, which is found across all other populations. However, this is in partial agreement with the results of our study, in which all isolates that possessed the *fosB* gene belonged only to clade A, a subclade of Trans Eurasia, but not to clade B. Similar results to ours were also reported by Chiaverini et al. [67]. 

Pathogenicity-related plasmids, pxO1 and pXO2, were found in all 40 *B. anthracis* isolates in this study. In addition, all isolates possessed the *atxA* gene, which is localised on the pathogenicity island pXO1 and is responsible for the expression of the three toxin genes [68,69].

Other virulence genes found in all *B. anthracis* strains in this study were *inhA1*, *inhA2*, *entFM*, *nheABC* operon, *plcR*, *sph*, *plcA*, and *alo.*

InhA1 cleaves multiple host proteins, including collagen, fibrinogen, plasminogen activator inhibitor, and prothrombin [70,71]. It also downregulates BslA, a *B. anthracis* cell surface protein associated with the adhesion of vegetative bacterial cells to the host endothelium [72]. The *B. anthracis* strains in this study also contained a gene encoding the metalloprotease InhA2, although it is not known whether this protease is expressed and secreted. This gene is an orthologue of InhA1 described above (68% amino acid identity) [73]. The metalloprotease (*inhA*) and the phospholipases *sph* genes are important virulence factors as they enable the bacilli to evade innate and adaptive immune responses during the infective phases [74,75].

The chromosomally encoded nheABC operon was previously described in all *B. anthracis* isolates [76]. It is responsible for the synthesis of termolabile non-haemolytic enterotoxin, which, together with enterotoxin FM and haemolysin HII, is involved in the diarrhoeic syndrome [77,78,79].

In this study, we used the cgMLST method to genotype *B. anthracis* isolates from the recent two decades in Croatia. The development of a cgMLST scheme for the entire *B. cereus* group is timely and ensures high-resolution strain typing of *B. cereus* group isolates in an open and fully publicly accessible online user interface for the future [80]. The first application of a whole-genome allele typing system was presented by Abdel-Glil et al. [29]. We used cgMLST-searching 3803 loci and accessory cgMLST-searching 1263 loci. After phylogenetic analysis based on the cgMLST data neighbour-joining tree was constructed (Figure 1). The colours represent the clade and subclade, which are also indicated by name, while the node labels correspond to the names of the strains and the country of origin. As shown in Figure 1, the Croatian *B. anthracis* isolates were categorised into two main clades, depending on the outbreaks: A and B. isolates from the 2006/2007 and 2022 outbreaks were categorised in clade B, subclade B2, while the isolates from the 2001/2002 outbreak and isolates from sporadic cases from the southern part of Croatia (ST2011) were categorised in clade A, subclade Trans Eurasia (TEA). The existence of two different genotypes of *B. anthracis* isolates in Croatia was also indicated by the finding that the *fosB* gene was discovered in all isolates from the 2001/2002 outbreak and in isolates from sporadic outbreaks from southern Croatia, while it was not present in isolates from the 2006/2007 and 2022 outbreaks. 

It has already been reported that strains belonging to clade A are responsible for the majority of anthrax cases reported worldwide, while the TEA subclade is one of the most common. These isolates are widespread in Europe, the Middle East, Russia, and the Chinese province of Xinjang [27,81]. In Europe, it is the most dominant subclade in Albania, Bulgaria, Hungary, and Italy [27,82]. The MLVA analysis of 234 *B. anthracis* strains isolated in Italy between 1972 and 2018 revealed the distribution of 55 *B. anthracis* genotypes, while the CanSNPs analysis categorised 53 of them into a TEA subclade [83]. As can be seen in Figure 1, the Croatian isolates assigned to the TEA subclade showed the greatest homology with the Italian isolate (3IZSLT).

Clade B occurs mainly in South Africa and in the Central Europe, especially in France and Poland. The South African strains mainly belong to the B1 subclade (B.Br.Kruger and B.Br.001/002 groups) [84], while the European strains belong to the B2 subclade (B.Br.CNEVA) [85,86]. Examples of these subclades are rarely found outside these regions [27]. The B2 subclade is widespread in Europe in south-west France, north-east Poland, northern Italy, Switzerland, Germany, and Slovenia, and it is the most dominant subclade in France, Poland, and northern Italy [87,88,89]. The Croatian *B. anthracis* isolates belonging to subclade B2 showed the most similarities with the Austrian isolates (Tyrol 3520) and, as can be seen in Figure 1, the entire B2 subclade branches from isolate ‘RA3 France’. Similar results were also obtained by Chiaverini et al. in Italy [67].

The TEA subclade has been found to occur in countries where the B2 subclade is dominant and is associated with sporadic cases [90], which is in complete agreement with our study.

These data show that anthrax outbreaks in Croatia are limited to a relatively small area of the country and are caused by two genetically very different *B. anthracis* genotypes belonging to two different, phylogenetically unrelated clades, indicating a different origin of the isolates.

## 4. Materials and Methods

### 4.1. Epidemiological Investigation

**Figure 2:**
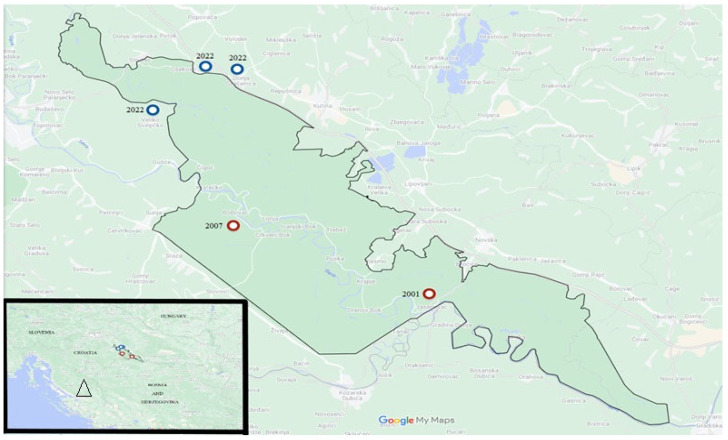
Geographical location of the Lonjsko Polje Nature Park. The locations of the 2022 outbreak are marked with blue dots. The red dots represent the anthrax outbreaks of 2001 and 2007. The triangle represents the southern part of Croatia where anthrax occurs sporadically.

At the end of June 2022, cattle breeders noticed an increase in cattle deaths at the Osekovo, Donja Gračenica, and Veliko Svinjičko pastures in the Lonjsko Polje Nature Park area in Croatia (Figure 2).

Considering the fact that it was very close to the border of the anthrax district, the Veterinary and Food Safety Authority authorised the dissection of the two carcasses. The first dissection of non-autolytic cattle was carried out on 15 July 2022 at the rendering plant under controlled conditions and the samples (blood sample, spleen) were delivered to the Laboratory of General Bacteriology and Mycology of the Croatian Veterinary Institute on the same day, as it was suspected that the animals had died of anthrax. Since the beginning of the outbreak of the disease until the end of 2022, 323 animals (246 cattle, 77 horses) have died in the Lonjsko Polje Nature Park area. This number is probably even higher, as we only have data on the number of dead animals brought to the rendering centre. 

By the end of 2022, a total of 101 necropsies were performed, mainly on animals that died on the same day or at least the day before. The autopsy of the corpses revealed that there was no rigour mortis and that unclogged blood leaked from all natural orifices. Oedema was found in the subcutaneous tissue. Splenomegaly, haemorrhages in the epicardium and endocardium, and pulmonary oedema with haemorrhages in the parenchyma were noted. The lymph nodes were enlarged and oedematous, and haemorrhages were observed in the parenchyma. Ulcerations were found on the mucosa of the abomasum and small intestine. Petechial haemorrhages were found in the serosa of the intestine.

**Figure 3:**
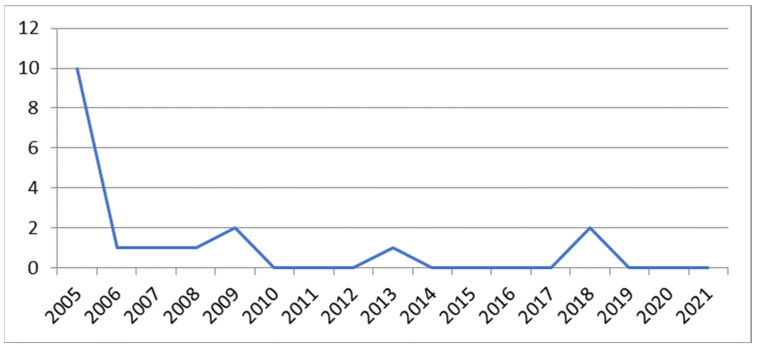
The timeline frequency of human anthrax in Croatia from 2005 to 2021.

Anthrax in humans in the Republic of Croatia is characterised by the occurrence of isolated cases. According to data from the Croatian Agency for Agriculture and Food, 16 cases of the disease were recorded in the Republic of Croatia in the period from 2005 to 2016 [91], while, according to data from the Croatian Institute of Public Health [92], two cases of the disease were recorded in humans in the period from 2017 to 2021 (in 2018). Figure 3. 

**Figure 4:**
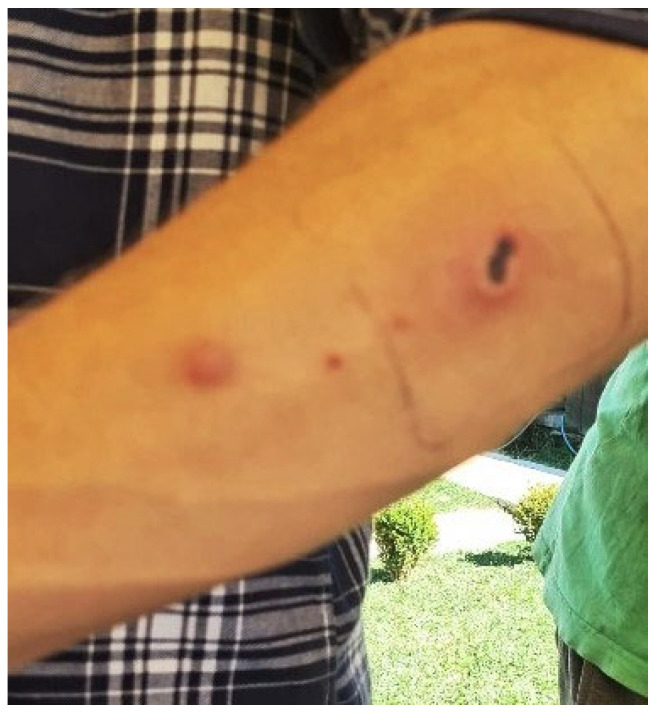
Cutaneous form of anthrax on human forearm.

The anthrax epidemic in humans in the Lonjsko Polje Nature Park began on 13 July 2022 and ended on 19 August 2022. Seventeen people fell ill with the cutaneous form of anthrax (Figure 4). Among the infected were 3 children up to the age of 8, 4 aged 15–18, and 10 adults. All had contact with sick cattle and were treated with antibiotics [93]. 

### 4.2. Laboratory Diagnosis

Fresh samples (mainly blood, spleen, and ears, with parts of the musculature) were sent to the Laboratory for General Bacteriology and Mycology of the Croatian Veterinary Institute. The spleen samples and ears with muscles were delivered in sterile plastic containers with screw caps, with each container being wrapped in two plastic bags to prevent leakage. The blood samples were delivered in syringes, and each syringe was placed in a plastic container with a screw cap.

Thin smears were made from all samples supplied and a Giemsa stain was used. The samples were inoculated onto blood agar (OXOID blood agar base No. 2 + 5% defibrinated sheep blood). After incubation under aerobic conditions at a temperature of 37 °C for 18–24 h, the plates were examined to determine bacterial growth, morphology, and colour of the bacterial colonies. Bacterial colonies morphologically consistent with *B. anthracis* were resuspended in distilled water and heated to 95 °C for 20 min. After cooling and centrifugation, the supernatant was used for a PCR reaction to confirm the presence of plasmids pXO1 and pXO2, using the previously described method [94,95]. 

The presence of plasmids pX01 and pX02 was confirmed in all *B. anthracis* strains from this epizootic as well as in the other strains from previous outbreaks in Croatia.

### 4.3. Isolates Used in This Study

We analysed a total of 40 *B. anthracis* isolates: 5 isolates from the 2001/2002 outbreak, 6 isolates from the 2006/2007 outbreak, 4 isolates from a southern part of Croatia, and 25 isolates from the 2022 outbreak. The molecular part of this study included the *B. anthracis* Sterne 34F2 (pXO1+, pXO2−) strain.

### 4.4. Antimicrobial Susceptibility Testing

Antimicrobial susceptibility testing (AST) was performed using the broth microdilution method according to Clinical and Laboratory Standards Institute (CLSI) guidelines [96].

Briefly, isolates were recovered from freezer stocks (tryptic soya broth with 20% glycerol, −80 °C) and incubated overnight on blood agar supplemented with 5% sheep blood in an aerobic atmosphere. AST was performed on GPN3F microtiter plates (Sensititer, Trek Diagnostic Systems Ltd. East Grinstead, West Sussex, UK). Cation-adjusted Mueller–Hinton broth was used to prepare the 0.5 McFarland solution. The microtiter plates were incubated in sealed 96-well microtiter plates at 35 ± 1 °C for 18 ± 2 h.

The susceptibility to erythromycin (ERY; 0.25–4 mg/L), quinupristin/dalfopristin (SYN; 0.12–4 mg/L), vancomycin (VAN; 1–128 mg/L), ampicillin (0.12–16 mg/L), rifampin (0.5–4 mg/L), levofloxacin (0.25–8 mg/L), penicillin (0.06–8 mg/L), trimethoprim/sulfamethoxazole (0.5/9.5–4/76 mg/L), oxacillin + 2%NaCl (0.25–8), ceftriaxone (8–64 mg/L), clindamycin (0.12–2 mg/L), daptomycin (0.25–8 mg/L), linezolid (0.5–8 mg/L), tetracycline (TET 2–16 mg/L), gentamicin (2–16 mg/L), gatifloxacin (1–8 mg/L), ciprofloxacin (0.5–2 mg/L), and streptomycin (1000 mg/L) was tested.

The lowest concentration of an antibiotic that prevented bacterial growth was taken as the minimum inhibitory concentration (MIC).

For penicillin, ampicillin, tetracycline, ciprofloxacin, and levofloxacin, the reported CLSI breakpoints for *B. anthracis* were used; for ceftriaxone, vancomycin, gentamicin, erythromicin, clindamycin, trimethoprim/sulfamethoxazole, and rifampin, Bacillus spp. breakpoints were used [96]. Interpretation criteria for *Staphylococcus* sp. were used for quinupristin/dalfopristin, oxacillin + 2%NaCl, linezolid, and gatifloxacin [97].

Streptomycin was not considered further, as there was only one concentration of this antimicrobial agent on the microplate, which was not important for further consideration.

The reference strains *Staphylococcus aureus* ATCC 29213 and *Escherichia coli* ATCC 25922 were used for quality control.

### 4.5. Whole-Genome Sequencing (WGS) and De Novo Assembly

The *B. anthracis* strains were sequenced using Illumina MiSeq (Illumina, San Diego, CA, USA). DNA was extracted using the Nucleospin Microbial DNA Kit (Macherey-Nagel, Düren, Germany). Paired-end sequencing libraries were prepared using the DNA LP Tagmentation kit (Illumina, USA), and the sequencing was performed using the MiSeq Reagent Kit v2 (300 cycles) (Illumina, USA). The raw sequences were trimmed with Trimmomatic v. 0.38.1. (options SLIDINGWINDOW 4, quality 20; MINLEN 100; LEADING 15; TRAILING 15; HEADCROP 15; AVGQUAL 25) [98]. Genome assembly was performed with Shovill v.1.1.0 (DEPTH 100; GENOME SIZE 5,5 Mb; POSTASSEMBLY CORRECTION 500; MIN COVERAGE 5) [99] for paired-end Illumina data. The assembly statistics were obtained using the program Quast v.5.0.2 [100].

The genome assemblies were deposited in GenBank under the BioProject accession number PRJNA1129309 (SAMN42150189—2001/2002 outbreak; SAMN42150190—2006/2007 outbreak; SAMN42150191—isolates from southern Croatia; SAMN42150192—2022 outbreak).

### 4.6. Bioinformatic Analysis of Antimicrobial Resistance and Virulence Genes

Virulence was tested with the VFDB database [101]. NCBI AMRFinderPlus v.3.11.26 was used for the identification of the resistance and virulence genes using BLASTX search [102]. MobileElementFinder v.1.0.5 was used for the identification of Mobile Genetic Elements (MGE) [103].

### 4.7. Core-Genome-Based Multilocus Sequence Typing (cgMLST)

cgMLST typing was performed using Ridom SeqSphere+ v.10.0.0 [104]. Both cgMLST-searching 3803 loci and accessory cgMLST-searching 1263 loci were used. For all analysed genomes, the cgMLST allelic profiles were pairwise-compared (missing values were ignored), and the resultant calculated pairwise distances were used to generate a neighbour-joining tree using SeqSphere+.

## 5. Conclusions

Anthrax in animals and humans in Croatia is characterised by sporadic outbreaks, which are limited to anthrax districts located mainly in the Lonjsko Polje region and in the southern parts of Croatia.

Rapid and reliable diagnosis and administration of antimicrobials are essential for PEP or effective treatment of anthrax. Molecular diagnosis, based on the presence of pXO1 and pXO2 plasmids and the morphological appearance of colonies, enables reliable anthrax diagnosis within 24 h in material that is not heavily contaminated.

Isolates of *B. anthracis* isolated in Croatia in the recent two decades were susceptible to all antimicrobials recommended for PEP or anthrax therapy (penicillin, ampicillin, tetracycline, and ciprofloxacin). Susceptibility was observed for all other antimicrobials tested, which represent an alternative for primary therapy. Three or four AMR genes were found in our isolates, depending on the area and year of the outbreak, of which two beta-lactamase genes were not expressed. In all isolates used in this study, we found 21 virulence genes, 8 of which are responsible for toxin and capsule production.

As far as phylogenetic analysis is concerned, the *B. anthracis* isolates from the outbreaks in Croatia are divided into two clades. The first is clade A, subclade Trans Eurasia, which is predominant in the Mediterranean region and the eastern part of Europe and was responsible for the 2001/2002 outbreak and the outbreaks in the southern part of the country. Isolates from the 2006/2007 and 2022 outbreaks are categorised in clade B, subclade B2, which is dominant in Central European countries. These results indicate that two different *B. anthracis* clades are circulating in a very small area, which is probably due to the geographical location of Croatia. 

## Figures and Tables

**Table 1:** PCR confirmation of pXO1 and pXO2 plasmids in 40 strains tested in Croatia.

Isolates	Number of Isolates	pXO1	pXO2
2001/2002	5	+	+
2006/2007	6	+	+
2022	25	+	+
ST	4	+	+
Sterne 34F2	1	+	-

**Table 2:** Antimicrobial susceptibility of 40 *B. anthracis* isolates from Croatia.

Antibiotic	MIC_50_	MIC_90_	Range (mg/L)	Breakpoint		% S/I/R ^a^
				S (≤)	R (≥)	
Erythromycin **	0.5	0.5	0.25–4	0.5	8	95/5/0
Quinupristin/dalfopristin ***	1	1	0.12–4	1	4	100/0/0
Vancomycin **	2	2	1–128	4		100/0/0
Ampicillin *	≤0.12	≤0.12	0.12–16	0.12	0.25	100/0/0
Rifampin **	≤0.5	≤0.5	0.5–4	1	4	100/0/0
Levofloxacin *	≤0.25	≤0.25	0.25–0.5	0.25		100/0/0
Penicillin *	≤0.06	≤0.06	0.06–8	0.5	1	100/0/0
Trimethoprim/sulfamethoxazole **	>4/76	>4/76	0.5/9.5–4/76	2/38	4/76	0/0/100
Oxacillin + 2%NaCl ***	≤0.25	≤0.25	0.5–8	0.5	1	100/0/0
Ceftriaxone **	≤8	16	8–64	8	64	60/40/0
Clindamycin **	0.25	0.25	0.12–2	0.5	4	100/0/0
Daptomycin ***	4	4	0.25–8	1		0/0/100
Linezolid ***	1	1	0.5–8	4	8	100/0/0
Tetracycline *	≤2	≤2	2–16	1		100/0/0
Gentamicin **	≤2	≤2	2–16	4	16	100/0/0
Gatifloxacin ***	≤1	≤1	1–8	0.5	2	100/0/0
Ciprofloxacin *	≤0.5	≤0.5	0.5–2	0.25		100/0/0
Streptomycin	≤1000	≤1000	1000			

^a^ S/I/R, susceptible/intermediate/resistant. * CLSI M45 *B. anthracis* interpretation. ** CLSI M45 *Bacillus* sp. interpretation. *** CLSI M100 *Staphylococcus* spp. interpretation.

**Table 3:** AMR and virulence factors genes of 40 *B. anthracis* isolates from Croatia.

Gene	Name	Type	Aligned Overlap 100%/Identity ≥ 97%			
			2001/2002 N = 5	2006/2007 N = 6	ST N = 4	2022 N = 25
*bla*	Class A beta-lactamase Bla1	AMR	+	+	+	+
*blaII*	BcII family subclass B1 metallo-beta-lactamase	AMR	+	+	+	+
*fosB*	fosB/fosD family fosfomycin resistance bacillithiol transferase	AMR	+	-	+	-
*satA*	Streptothricin N- acetyltransferase SatA	AMR	+	+	+	+
*pagA*	anthrax toxin protective antigen	VIR	+	+	+	+
*lef*	anthrax toxin lethal factor	VIR	+	+	+	+
*cya*	anthrax toxin edema factor	VIR	+	+	+	+
*capA*	capsular polyglutamate synthetase CapA	VIR	+	+	+	+
*capB*	capsule biosynthesis protein CapB	VIR	+	+	+	+
*capC*	capsular polyglutamate amide ligase/translocase CapC	VIR	+	+	+	+
*capD*	capsule biosynthesis gamma-glutamyltransferase CapD	VIR	+	+	+	+
*capE*	capsule biosynthesis protein CapE	VIR	+	+	+	+
*atxA*	anthrax toxin expression trans-acting transcriptional regulator AtxA	VIR	+	+	+	+
*inhA1*	M6 family metalloprotease immune inhibitor InhA1	VIR	+	+	+	+
*inhA2*	M6 family metalloprotease immune inhibitor InhA2	VIR	+	+	+	+
*entFM*	enterotoxin EntFM	VIR	+	+	+	+
*nheA*	non-hemolytic enterotoxin NHE subunit A	VIR	+	+	+	+
*nheB*	non-hemolytic enterotoxin NHE subunit B	VIR	+	+	+	+
*nheC*	non-hemolytic enterotoxin NHE subunit C	VIR	+	+	+	+
*plcR*	transcriptional regulator PlcR	VIR	+	+	+	+
*sph*	sphingomyelinase C	VIR	+	+	+	+
*hlyIIR*	hemolysin II regulator HlyIIR	VIR	+	+	+	+
*cerA*	phospholipase CerA	VIR	+	+	+	+
*plcA*	phosphatidylinositol diacylglycerol-lyase	VIR	+	+	+	+
*alo*	anthrolysin O/cereolysin O family cholesterol-dependent cytolysin Alo	VIR	+	+	+	+

AMR—antimicrobial resistance gene. VIR—virulence factor gene.

## Data Availability

The data are contained in the article and Supplementary Materials.

## Supplementary Materials

The following supporting information can be downloaded at: https://www.mdpi.com/article/10.3390/antibiotics13070639/s1, File S1: Historical review of anthrax in Croatia. References [105,106,107,108,109] are cited in the Supplementary Materials.

## References

[1] Liu Y., Lai Q., Göker M., Meier-Kolthoff J.P., Wang M., Sun Y., Wang L., Shao Z. (2015). Genomic insights into the taxonomic status of the *Bacillus cereus* group. Sci. Rep..

[2] Swartz M.N. (2001). Recognition and management of anthrax—An update. N. Engl. J. Med..

[3] McKendrick D.R. (1980). Anthrax and its transmission to humans. Cent. Afr. J. Med..

[4] Kiple K.F. (1993). The Cambridge World History of Human Disease.

[5] Ringertz S.H., Høiby E.A., Jensenius M., Maehlen J., Caugant D.A., Myklebust A., Fossum K. (2000). Injectional anthrax in a heroin skin-popper. Lancet.

[6] Jernigan J.A., Stephens D.S., Ashford D.A., Omenaca C., Topiel M.S., Galbraith M., Tapper M., Fisk T.L., Zaki S., Popovic T. (2001). Anthrax Bioterrorism Investigation Team. Bioterrorism-related inhalational anthrax: The first 10 cases reported in the United States. Emerg. Infect Dis..

[7] Fowler R.A., Shafazand S. (2011). Anthrax Bioterrorism: Prevention, Diagnosis and Management Strategies. J. Bioterr. Biodef..

[8] World Health Organization, Food and Agriculture Organization of the United Nations, World Organisation for Animal Health (2008). Anthrax in Humans and Animals.

[9] Jacotot H., Virat B. (1954). Long duration of viability of spores of *Bacillus anthracis*. Ann. Inst. Pasteur.

[10] Wilson J.B., Russell K.E. (1964). Isolation of *Bacillus anthracis* from soil stored 60 years. J. Bacteriol..

[11] De Vos V. (1990). The ecology of anthrax in the Kruger National Park, South Africa. Salisbury Med. Bull..

[12] Sterne M. (1937). The effects of different carbon dioxide concentrations on the growth of virulent anthrax strains. Pathogenicity and immunity tests on guinea pigs and sheep with anthrax variants derived from virulent strains. Onderstepoort J. Vet. Sci. Anim. Ind..

[13] Sterne M. (1939). The use of anthrax vaccines prepared from avirulent (uncapsulated) variants of *Bacillus anthracis*. Onderstepoort J. Vet. Sci. Anim. Ind..

[14] Mikesell P., Ivins B.E., Ristroph J.D., Dreier T.M. (1983). Evidence for plasmid-mediated toxin production in *Bacillus anthracis*. Infect. Immun..

[15] Okinaka R., Cloud K., Hampton O., Hoffmaster A., Hill K., Keim P., Koehler T., Lamke G., Kumano S., Manter D. (1999). Sequence, assembly and analysis of pX01 and pX02. J. Appl. Microbiol..

[16] Young J.A., Collier R.J. (2007). Anthrax toxin: Receptor binding, internalization, pore formation, and translocation. Annu. Rev. Biochem..

[17] Candela T., Fouet A. (2006). Poly-gamma-glutamate in bacteria. Mol. Microbiol..

[18] Green B.D., Battisti L., Koehler T.M., Thorne C.B., Ivins B.E. (1985). Demonstration of a capsule plasmid in *Bacillus anthracis*. Infect. Immun..

[19] Henderson I., Duggleby C.J., Turnbull P.C. (1994). Differentiation of *Bacillus anthracis* from other *Bacillus cereus* group bacteria with the PCR. Int. J. Syst. Bacteriol..

[20] Klee S.R., Ozel M., Appel B., Boesch C., Ellerbrok H., Jacob D., Holland G., Leendertz F.H., Pauli G., Grunow R. (2006). Characterization of *Bacillus anthracis*-like bacteria isolated from wild great apes from Cote d’Ivoire and Cameroon. J. Bacteriol..

[21] Rasko D.A., Rosovitz M.J., Økstad O.A., Fouts D.E., Jiang L., Cer R.Z., Kolstø A.B., Gill S.R., Ravel J. (2007). Complete sequence analysis of novel plasmids from emetic and periodontal *Bacillus cereus* isolates reveals a common evolutionary history among the B. cereus-group plasmids, including *Bacillus anthracis* pXO1. J. Bacteriol..

[22] Van der Auwera G.A., Andrup L., Mahillon J. (2005). Conjugative plasmid pAW63 brings new insights into the genesis of the Bacillus anthracis virulence plasmid pXO2 and of the *Bacillus thuringiensis* plasmid pBT9727. BMC Genom..

[23] Keim P., Price L.B., Klevytska A.M., Smith K.L., Schupp J.M., Okinaka R., Jackson P.J., Hugh-Jones M.E. (2000). Multiple-locus variable-number tandem repeat analysis reveals genetic relationships within *Bacillus anthracis*. J. Bacteriol..

[24] Keim P., Gruendike J.M., Klevytska A.M., Schupp J.M., Challacombe J., Okinaka R. (2009). The genome and variation of *Bacillus anthracis*. Mol. Aspects Med..

[25] Kolstø A.B., Tourasse N.J., Økstad O.A. (2009). What sets *Bacillus anthracis* apart from other *Bacillus* species?. Annu. Rev. Microbiol..

[26] Andersen G.L., Simchock J.M., Wilson K.H. (1996). Identification of a region of genetic variability among *Bacillus anthracis* strains and related species. J. Bacteriol..

[27] Van Ert M.N., Easterday W.R., Huynh L.Y., Okinaka R.T., Hugh-Jones M.E., Ravel J., Zanecki S.R., Pearson T., Simonson T.S., U’Ren J.M. (2007). Global genetic population structure of *Bacillus anthracis*. PLoS ONE.

[28] Thierry S., Tourterel C., Le Flèche P., Derzelle S., Dekhil N., Mendy C., Colaneri C., Vergnaud G., Madani N. (2014). Genotyping of French *Bacillus anthracis* strains based on 31-loci multi locus VNTR analysis: Epidemiology, marker evaluation, and update of the internet genotype database. PLoS ONE.

[29] Abdel-Glil M.Y., Chiaverini A., Garofolo G., Fasanella A., Parisi A., Harmsen D., Jolley K.A., Elschner M.C., Tomaso H., Linde J. (2021). A Whole-Genome-Based Gene-by-Gene Typing System for Standardized High-Resolution Strain Typing of *Bacillus anthracis*. J. Clin. Microbiol..

[30] Sahl J.W., Pearson T., Okinaka R., Schupp J.M., Gillece J.D., Heaton H., Birdsell D., Hepp C., Fofanov V., Noseda R. (2016). A *Bacillus anthracis* Genome Sequence from the Sverdlovsk 1979 Autopsy Specimens. mBio.

[31] Habrun B., Račić I., Kompes G., Špičić S., Benić M., Mihaljević Ž., Cvetnić Ž. (2011). The antimicrobial susceptibility and virulence factors of *Bacillus anthracis* strains isolated in Croatia. Vet. Med..

[32] Ministry of Agriculture of the Republic of Croatia. http://www.veterinarstvo.hr/default.aspx?id=185.

[33] Šoštarić B., Habrun B., Lipej Z., Vicković I. (2002). Prikaz Epizootije Bedrenice u Stadu Ovaca i Koza. Veterinarski Dani, Zagreb, Hrvatska, 17.10.2002–20.10.2002.

[34] Habrun B., Kompes G., Šeol B. (2009). Anthrax—Epizootic case in Bobovac (Sunja). Vet. Stanica.

[35] Miškić T., Lohman Janković I. (2023). Epizootiološke Značajke Bedrenice na Području Parka Prirode Lonjsko Polje 2022. Sažetci, BEDRENICA I AFRIČKA SVINJSKA KUGA U HRVATSKOJ, Zagreb, Hrvatska, 13.10.2023.

[36] Schmid G., Kaufmann A. (2002). Anthrax in Europe: Its epidemiology, clinical characteristics, and role in bioterrorism. Clin. Microbiol. Infect..

[37] Nicastri E., Vairo F., Mencarini P., Battisti A., Agrati C., Cimini E., Carrara S., D’Arezzo S., Adone R., Vulcano A. (2019). Unexpected human cases of cutaneous anthrax in Latium region, Italy, August 2017: Integrated human-animal investigation of epidemiological, clinical, microbiological and ecological factors. Euro Surveill..

[38] Hodnik J.J., Knific T., Starič J., Toplak I., Ocepek M., Hostnik P., Ježek J. (2021). Overview of Slovenian Control Programmes for Cattle Diseases Not Regulated by the European Union. Front. Vet. Sci..

[39] Orlos Z., Rakoczi E., Misak O., Lenart B., Ocsai G., Kovacs I., Gorzsas S., Kardos L., Lampe Z., Szilvassy Z. (2017). Outbreak of anthrax in adults and adolescents: A review of nine cases in a regional teaching hospital in East Hungary. Clin. Microbiol. Infect..

[40] Arapović J., Skočibusić S., Jelavić B., Ivanković H.B., Jurić M., Mamić D., Grgić S., Lesko J., Leventić M., Soldo I. (2015). Two cases of human cutaneous anthrax in Bosnia and Herzegovina, September 2014. Euro Surveill..

[41] Bower W.A., Yu Y., Person M.K., Parker C.M., Kennedy J.L., Sue D., Hesse E.M., Cook R., Bradley J., Bulitta J.B. (2023). CDC Guidelines for the Prevention and Treatment of Anthrax 2023. MMWR Recomm Rep..

[42] Severn M. (1976). A fatal case of pulmonary anthrax. Br. Med. J..

[43] Cavallo J.D., Ramisse F., Girardet M., Vaissaire J., Mock M., Hernandez E. (2002). Antibiotic susceptibilities of 96 isolates *of Bacillus anthracis* isolated in France between 1994 and 2000. Antimicrob. Agents Chemother..

[44] Maxson T., Kongphet-Tran T., Mongkolrattanothai T., Travis T., Hendricks K., Parker C., McLaughlin H.P., Bugrysheva J., Ambrosio F., Michel P. (2022). Systematic Review of In Vitro Antimicrobial Susceptibility Testing for *Bacillus anthracis*, 1947–2019. Clin. Infect. Dis..

[45] Manzulli V., Fasanella A., Parisi A., Serrecchia L., Donatiello A., Rondinone V., Caruso M., Zange S., Tscherne A., Decaro N. (2019). Evaluation of in vitro antimicrobial susceptibility of *Bacillus anthracis* strains isolated during anthrax outbreaks in Italy from 1984 to 2017. J. Vet. Sci..

[46] Chen Y., Succi J., Tenover F.C., Koehler T.M. (2003). Beta-lactamase genes of the penicillin-susceptible *Bacillus anthracis* Sterne strain. J. Bacteriol..

[47] Materon I.C., Queenan A.M., Koehler T.M., Bush K., Palzkill T. (2003). Biochemical characterization of beta-lactamases Bla1 and Bla2 from *Bacillus anthracis*. Antimicrob. Agents Chemother..

[48] Read T.D., Peterson S.N., Tourasse N., Baillie L.W., Paulsen I.T., Nelson K.E., Tettelin H., Fouts D.E., Eisen J.A., Gill S.R. (2003). The genome sequence of *Bacillus anthracis* Ames and comparison to closely related bacteria. Nature.

[49] Slamti L., Perchat S., Gominet M., Vilas-Bôas G., Fouet A., Mock M., Sanchis V., Chaufaux J., Gohar M., Lereclus D. (2004). Distinct mutations in PlcR explain why some strains of the *Bacillus cereus* group are nonhemolytic. J. Bacteriol..

[50] Gohar M., Økstad O.A., Gilois N., Sanchis V., Kolstø A.B., Lereclus D. (2002). Two-dimensional electrophoresis analysis of the extracellular proteome of *Bacillus cereus* reveals the importance of the PlcR regulon. Proteomics.

[51] Agaisse H., Gominet M., Okstad O.A., Kolstø A.B., Lereclus D. (1999). PlcR is a pleiotropic regulator of extracellular virulence factor gene expression in *Bacillus thuringiensis*. Mol. Microbiol..

[52] Sastalla I., Maltese L.M., Pomerantseva O.M., Pomerantsev A.P., Keane-Myers A., Leppla S.H. (2010). Activation of the latent PlcR regulon in *Bacillus anthracis*. Microbiology.

[53] Ross C.L., Thomason K.S., Koehler T.M. (2009). An extracytoplasmic function sigma factor controls beta-lactamase gene expression in *Bacillus anthracis* and other *Bacillus cereus* group species. J. Bacteriol..

[54] Ho T.D., Nauta K.M., Müh U., Ellermeier C.D. (2019). Activation of the Extracytoplasmic Function σ Factor σP by β-Lactams in *Bacillus thuringiensis* Requires the Site-2 Protease RasP. mSphere.

[55] Jones M.E., Goguen J., Critchley I.A., Draghi D.C., Karlowsky J.A., Sahm D.F., Porschen R., Patra G., DelVecchio V.G. (2003). Antibiotic susceptibility of isolates of *Bacillus anthracis*, a bacterial pathogen with the potential to be used in biowarfare. Clin. Microbiol. Infect..

[56] Luna V.A., King D.S., Gulledge J., Cannons A.C., Amuso P.T., Cattani J. (2007). Susceptibility of *Bacillus anthracis*, *Bacillus cereus*, *Bacillus mycoides*, *Bacillus pseudomycoides* and *Bacillus thuringiensis* to 24 antimicrobials using Sensititre automated microbroth dilution and Etest agar gradient diffusion methods. J. Antimicrob. Chemother..

[57] Maho A., Rossano A., Hächler H., Holzer A., Schelling E., Zinsstag J., Hassane M.H., Toguebaye B.S., Akakpo A.J., Van Ert M. (2006). Antibiotic susceptibility and molecular diversity of *Bacillus anthracis* strains in Chad: Detection of a new phylogenetic subgroup. J. Clin. Microbiol..

[58] Swaney S.M., Aoki H., Ganoza M.C., Shinabarger D.L. (1998). The oxazolidinone linezolid inhibits initiation of protein synthesis in bacteria. Antimicrob. Agents Chemother..

[59] Pomerantsev A.P., Sukovatova L.V., Marinin L.I. (1993). Izuchenie svoĭstv Rif-R-populiatsii sibireiazvennogo mikroba [Characterization of a Rif-R population of *Bacillus anthracis*]. Antibiot. I Khimioterapiia Antibiot. Chemoter..

[60] Kim H.S., Choi E.C., Kim B.K. (1993). A macrolide-lincosamide-streptogramin B resistance determinant from *Bacillus anthracis* 590: Cloning and expression of ermJ. J. Gen. Microbiol..

[61] Sejvar J.J., Tenover F.C., Stephens D.S. (2005). Management of anthrax meningitis. Lancet Infect. Dis..

[62] Athamna A., Massalha M., Athamna M., Nura A., Medlej B., Ofek I., Bast D., Rubinstein E. (2004). In vitro susceptibility of *Bacillus anthracis* to various antibacterial agents and their time-kill activity. J. Antimicrob. Chemother..

[63] Odendaal M.W., Pieterson P.M., de Vos V., Botha A.D. (1991). The antibiotic sensitivity patterns of *Bacillus anthracis* isolated from the Kruger National Park. Onderstepoort J. Vet. Res..

[64] Heine H.S., Bassett J., Miller L., Purcell B.K., Byrne W.R. (2010). Efficacy of Daptomycin against *Bacillus anthracis* in a murine model of anthrax spore inhalation. Antimicrob. Agents Chemother..

[65] Burckhardt R.M., Escalante-Semerena J.C. (2019). Insights into the Function of the *N*-Acetyltransferase SatA That Detoxifies Streptothricin in *Bacillus subtilis* and *Bacillus anthracis*. Appl. Environ. Microbiol..

[66] Bruce S.A., Huang Y.H., Kamath P.L., van Heerden H., Turner W.C. (2021). The roles of antimicrobial resistance, phage diversity, isolation source and selection in shaping the genomic architecture of *Bacillus anthracis*. Microb. Genom..

[67] Chiaverini A., Abdel-Glil M.Y., Linde J., Galante D., Rondinone V., Fasanella A., Cammà C., D’Alterio N., Garofolo G., Tomaso H. (2020). Whole Genome Sequencing for Studying *Bacillus anthracis* from an Outbreak in the Abruzzo Region of Italy. Microorganisms.

[68] Uchida I., Hornung J.M., Thorne C.B., Klimpel K.R., Leppla S.H. (1993). Cloning and characterization of a gene whose product is a trans-activator of anthrax toxin synthesis. J. Bacteriol..

[69] Koehler T.M., Dai Z., Kaufman-Yarbray M. (1994). Regulation of the *Bacillus anthracis* protective antigen gene: CO_2_ and a trans-acting element activate transcription from one of two promoters. J. Bacteriol..

[70] Chung M.C., Popova T.G., Millis B.A., Mukherjee D.V., Zhou W., Liotta L.A., Petricoin E.F., Chandhoke V., Bailey C., Popov S.G. (2006). Secreted neutral metalloproteases of *Bacillus anthracis* as candidate pathogenic factors. J. Biol. Chem..

[71] Chung M.C., Jorgensen S.C., Popova T.G., Tonry J.H., Bailey C.L., Popov S.G. (2009). Activation of plasminogen activator inhibitor implicates protease InhA in the acute-phase response to *Bacillus anthracis* infection. J. Med. Microbiol..

[72] Tonry J.H., McNichol B.A., Ramarao N., Chertow D.S., Kim K.S., Stibitz S., Schneewind O., Kashanchi F., Bailey C.L., Popov S. (2012). *Bacillus anthracis* protease InhA regulates BslA-mediated adhesion in human endothelial cells. Cell Microbiol..

[73] Gomis-Rüth F.X. (2009). Catalytic domain architecture of metzincin metalloproteases. J. Biol. Chem..

[74] Ramarao N., Lereclus D. (2005). The InhA1 metalloprotease allows spores of the B. cereus group to escape macrophages. Cell Microbiol..

[75] Oda M., Hashimoto M., Takahashi M., Ohmae Y., Seike S., Kato R., Fujita A., Tsuge H., Nagahama M., Ochi S. (2012). Role of sphingomyelinase in infectious diseases caused by *Bacillus cereus*. PLoS ONE.

[76] Cai Y., Huang T., Xu Y., Zhou G., Zou P., Zeng G., Liu X. (2017). Genetic and genomic diversity of NheABC locus from *Bacillus* strains. Arch. Microbiol..

[77] Asano S.I., Nukumizu Y., Bando H., Iizuka T., Yamamoto T. (1997). Cloning of novel enterotoxin genes from *Bacillus cereus* and *Bacillus thuringiensis*. Appl. Environ. Microbiol..

[78] Boonchai N., Asano S.I., Bando H., Wiwat C. (2008). Study on cytotoxicity and nucleotide sequences of enterotoxin FM of *Bacillus cereus* isolated from various food sources. J. Med. Assoc. Thai..

[79] Castiaux V., Laloux L., Schneider Y.J., Mahillon J. (2016). Screening of Cytotoxic *B. cereus* on Differentiated Caco-2 Cells and in Co-Culture with Mucus-Secreting (HT29-MTX) Cells. Toxins.

[80] Tourasse N.J., Jolley K.A., Kolstø A.B., Økstad O.A. (2023). Core genome multilocus sequence typing scheme for *Bacillus cereus* group bacteria. Res. Microbiol..

[81] Simonson T.S., Okinaka R.T., Wang B., Easterday W.R., Huynh L., U’Ren J.M., Dukerich M., Zanecki S.R., Kenefic L.J., Beaudry J. (2009). *Bacillus anthracis* in China and its relationship to worldwide lineages. BMC Microbiol..

[82] Antwerpen M., Ilin D., Georgieva E., Meyer H., Savov E., Frangoulidis D. (2011). MLVA and SNP analysis identified a unique genetic cluster in Bulgarian *Bacillus anthracis* strains. Eur. J. Clin. Microbiol. Infect. Dis..

[83] Rondinone V., Serrecchia L., Parisi A., Fasanella A., Manzulli V., Cipolletta D., Galante D. (2020). Genetic characterization of *Bacillus anthracis* strains circulating in Italy from 1972 to 2018. PLoS ONE.

[84] Smith K.L., DeVos V., Bryden H., Price L.B., Hugh-Jones M.E., Keim P. (2000). *Bacillus anthracis* diversity in Kruger National Park. J. Clin. Microbiol..

[85] Fouet A., Smith K.L., Keys C., Vaissaire J., Le Doujet C., Lévy M., Mock M., Keim P. (2002). Diversity among French *Bacillus anthracis* isolates. J. Clin. Microbiol..

[86] Gierczyński R., Kałuzewski S., Rakin A., Jagielski M., Zasada A., Jakubczak A., Borkowska-Opacka B., Rastawicki W. (2004). Intriguing diversity of *Bacillus anthracis* in eastern Poland--the molecular echoes of the past outbreaks. FEMS Microbiol. Lett..

[87] Derzelle S., Laroche S., Le Flèche P., Hauck Y., Thierry S., Vergnaud G., Madani N. (2011). Characterization of genetic diversity of *Bacillus anthracis* in France by using high-resolution melting assays and multilocus variable-number tandem-repeat analysis. J. Clin. Microbiol..

[88] Garofolo G., Serrecchia L., Corrò M., Fasanella A. (2011). Anthrax phylogenetic structure in Northern Italy. BMC Res. Notes.

[89] Girault G., Blouin Y., Vergnaud G., Derzelle S. (2014). High-throughput sequencing of *Bacillus anthracis* in France: Investigating genome diversity and population structure using whole-genome SNP discovery. BMC Genom..

[90] Derzelle S., Thierry S. (2013). Genetic diversity of *Bacillus anthracis* in Europe: Genotyping methods in forensic and epidemiologic investigations. Biosecur. Bioterror..

[91] Croatian Food Agency. https://www.hzjz.hr/wp-content/uploads/2017/11/Godi%C5%A1nje-izvje%C5%A1%C4%87e-o-zoonozama-2015_16.pdf.

[92] Croatian Institute for Public Health (2022). Croatian Health Statistics Yearbook. https://www.hzjz.hr/wp-content/uploads/2024/05/HZSLj_2022_12-2023.pdf.

[93] Pajtlar S., Ančić-Birač S., Starčević M. (2023). Epidemija Antraksa u Parku Prirode Lonjsko Polje 2022, Sažetci, BEDRENICA I AFRIČKA SVINJSKA KUGA U HRVATSKOJ, Zagreb, Hrvatska, 13.10.2023.

[94] Beyer W., Glöckner P., Otto J., Böhm R. (1995). A nested PCR method for the detection of *Bacillus anthracis* in environmental samples collected from former tannery sites. Microbiol. Res..

[95] Hutson R.A., Duggleby C.J., Lowe J.R., Manchee R.J., Turnbull P.C. (1993). The development and assessment of DNA and oligonucleotide probes for the specific detection of *Bacillus anthracis*. J. Appl. Bacteriol..

[96] Clinical and Laboratory Standards Institute (CLSI) (2016). Methods for Antimicrobial Dilution and Disk Susceptibility Testing of Infrequently Isolated or Fastidious Bacteria M45.

[97] Clinical and Laboratory Standards Institute (CLSI) (2022). Performance Standards for Antimicrobial Susceptibility Testing, M100.

[98] Bolger A.M., Lohse M., Usadel B. (2014). Trimmomatic: A flexible trimmer for Illumina sequence data. Bioinformatics.

[99] Bankevich A., Nurk S., Antipov D., Gurevich A.A., Dvorkin M., Kulikov A.S., Lesin V.M., Nikolenko S.I., Pham S., Prjibelski A.D. (2012). SPAdes: A new genome assembly algorithm and its applications to single-cell sequencing. J. Comput. Biol..

[100] Gurevich A., Saveliev V., Vyahhi N., Tesler G. (2013). QUAST: Quality assessment tool for genome assemblies. Bioinformatics.

[101] Chen L., Zheng D., Liu B., Yang J., Jin Q. (2016). VFDB 2016: Hierarchical and refined dataset for big data analysis—10 years on. Nucleic Acids Res..

[102] Feldgarden M., Brover V., Haft D.H., Prasad A.B., Slotta D.J., Tolstoy I., Tyson G.H., Zhao S., Hsu C.H., McDermott P.F. (2019). Validating the AMRFinder Tool and Resistance Gene Database by Using Antimicrobial Resistance Genotype-Phenotype Correlations in a Collection of Isolates. Antimicrob. Agents Chemother..

[103] Johansson M.H.K., Bortolaia V., Tansirichaiya S., Aarestrup F.M., Roberts A.P., Petersen T.N. (2021). Detection of mobile genetic elements associated with antibiotic resistance in *Salmonella enterica* using a newly developed web tool: MobileElementFinder. J. Antimicrob. Chemother..

[104] Jünemann S., Sedlazeck F.J., Prior K., Albersmeier A., John U., Kalinowski J., Mellmann A., Goesmann A., von Haeseler A., Stoye J. (2013). Updating benchtop sequencing performance comparison. Nat. Biotechnol..

[105] Maestro M. (1946). Bedrenica Domaćih Životinja u BIVŠOJ SAVSKOJ BANOVINI od 1931–1940 Godine. Ph.D. Thesis.

[106] Anon (1888). Zakona o uređenju veterinarstva u Kraljevinah Hrvatskoj i Slavoniji. Nar. Nov. (Tisk. Zavod Zagreb).

[107] Mlinac F. (1930). Pojavljivanje Bedrenice na Području Bivše Hrvatske i Slavonije. Ph.D. Thesis.

[108] Zaharija I. (1978). Zarazne Bolesti Domaćih Životinja.

[109] Fališevac J., Hellenbach H. (1951). Antraks kao profesionalno oboljenje sa specijalnim osvrtom na prilike u Jugoslaviji. Arh. Hig. Rada Toksikol..

