# Supplementary material for: Antimicrobial Resistance Profile, Whole-Genome Sequencing and Core Genome Multilocus Sequence Typing of B. anthracis Isolates in Croatia from 2001 to 2022
Source: Antibiotics (Basel). 2024 Jul 11;13(7):639. doi: 10.3390/antibiotics13070639 (PMC11274125; doi:10.3390/antibiotics13070639)
Supplement: Supplementary file 1 [file antibiotics-13-00639-s001.zip › antibiotics-3086813-supplementary.pdf]

Supplemenatry Materials

File S1: Historical data on anthrax in humans and animals in Croatia

The Croatian territory, which was called Sava Banovina until 1939, comprised roughly the same borders as today's Croatia, with the exception of Istria and Dalmatia. The area was characterised by a large number of rivers (Danube, Drava, Mura, Una, Kupa, Lika, Gacka, Zrmanja), which flooded the surrounding fields, meadows and pastures when the water level rose during heavy rainfall. For this reason, the entire area of the former Sava Banovina was an anthrax area, as can be seen in Figure S1.

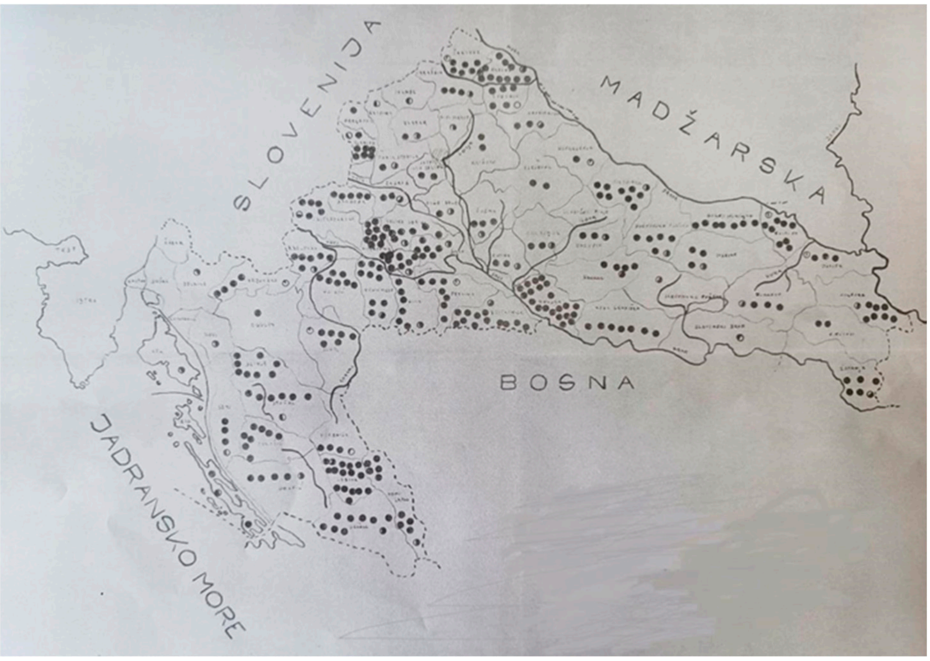

Figure S1. Morbidity from anthrax in Croatia from 1931 to 1940 [105]. Legend: the degree of filling of the circle represents a percentage of livestock mortality. Bosna – Bosnia and Herzegovina; Madžarska – Hungary; Slovenija – Slovenia; Jadransko more – Adriatic sea

Due to this situation, the first law was passed in 1988, which for the first time provided for measures to combat anthrax [106]. The first concrete data on anthrax cases in cattle in Croatia came from the doctoral thesis of F. Mlinac [107]. In the period from 1909 to 1923, 5164 cattle became ill from anthrax in Sava Banovina. Data on the number of dead animals for this period are not available. In the period from 1931 to 1940 [105], 8059 animals became ill from anthrax in Sava Banovina, while 5119 animals died. Morbidity due to anthrax in Croatia from 1931 to 1940 is shown in Figure 1. In the period from 1945 to 1959, 25 out of 100,000 animals in Croatia fell ill from anthrax, while 2445 animals died from 1947 to 1971 [108]. The first anthrax vaccine for animals was produced in Croatia in 1923 at the State Bacteriological and Serological Institute in Križevci. The high mortality rate that occurred even after the vaccination of animals encouraged Josip Ježić, PhD, DMV, to explore the possibility of improving the anthrax vaccine. The research was completed in 1933 with two improved vaccines: a disposable intracutaneous vaccine for cattle, sheep and pigs and a subcutaneous glucoside-saponin-based vaccine. In the period from 1934 to 1940, 474734 cattle, 34641 sheep and 91719 horses were vaccinated with these vaccines in Croatia, i.e. a total of 601094 animals. The number of anthrax cases systematically decreased after 1940, due to successful prophylaxis by vaccinating animals, the establishment of watercourses, flood defence measures, drainage and the construction of dams. At the end of the 20th and beginning of the 21st century, anthrax in Croatia occurred mostly sporadically, with one or two dead animals per year. The characteristics of human anthrax in Croatia were partially

illuminated by analysing 114 anthrax cases treated at the Infectious Diseases Hospital in Zagreb between 1936 and 1950. 113 cases were pure cutaneous anthrax with solitary or multiple malignant pustules and one patient had a combination of cutaneous and gastrointestinal forms of anthrax [109].
